# Meteorological Table

**Published:** 1811-01

**Authors:** 


					I 95 3
METEOROLOGICAL TABLE,
From Oct. 29, to Nov. 27.
D
28
29
SO
1
. 2
S 3
' 4
5
' 6
.7
8
9
.?10
11
12
13
14
15
16
17
IS
P19
20,
21!
22!
23
24
25
126
27
28
Hygrom.
dry-^?damp.
1042 31
34 36
38 35
85 ?
30 27
30 42
57 52
58 -
50 ?
48 40
40 36
35 38
40 50
47 35
? 38 55
43 ?
51 22
30 '27
?w-30 28
?32 48
54 52
35 34
35 ?
40 21
10 30
52 32
39 ?
55 29
21 17
52 18
24 10
Weather.
40| clear
cloudy .. rain ,
cloudy.? clear
clear ... ? . rain
30lclear
50
57
48!
4s!
clear
clear ... rain ... ??
fog... cloudy.. rain...
clear .. cloudy .. ?
rain .. cloudy.. rain.. clear.
clear .... ? ? ... ?
a... snow. cloudy .. rain.
cloudy .. .clear ....
rain . ? .. clear .. .
cloudy.clear...rain..inn. ...*
cloudy... clear. ? .. .*
clear... dry fog... clear... .*-
fog.. clear.. rain.. cloud...
cloudy ... rain... clear ...
clear... ? ... ??
clear.. .. rain . cloudy .. ?
cloudy .... rain. clear...
clear.... rain ... ? ..
rain . cloudy.. clear....
rain . .. clear .... ? .?
rain'.... clear, .hail ...cloud..
rain ... clear ... ? ?
'cloudy... snow . clear.. ?
13. Heavy fall of rain in the night of the 13th and morning of the 14th, witk
gale of wind from N.W.
14. Stormy wind N.W. and quick evaporation.
16. Dry fog passing in large masses, with intervals of sun.
21. Stormy wind from N. W. the whole day. Hygrometer rises rabidly t*
$he dry point: quick evaporation in the night.
25. Serene lightning from eleven to twelve at night.
frinrf('Strut, Gavendish-Syuari! Nov. 29,1810.

				

